# A Novel Attenuated Enterovirus A71 Mutant with VP1-V238A,K244R Exhibits Reduced Efficiency of Cell Entry/Exit and Augmented Binding Affinity to Sulfated Glycans

**DOI:** 10.1128/JVI.01055-21

**Published:** 2021-10-27

**Authors:** Tao Meng, Sek-Man Wong, Kaw-Bing Chua

**Affiliations:** a Temasek Life Sciences Laboratory, Limited, Republic of Singapore; b Department of Biological Sciences, National University of Singapore, Republic of Singapore; c NUS Suzhou Research Institute, Suzhou, People’s Republic of China; Instituto de Biotecnologia/UNAM

**Keywords:** enterovirus A71, cell-adapted mutants, live attenuated vaccines, sulfated glycans, virulence determinants, virus dissemination, virus entry/exit

## Abstract

Enterovirus A71 (EV-A71) is one of the major etiological agents of hand, foot, and mouth disease (HFMD), and infection occasionally leads to fatal neurological complications in children. However, only inactivated whole-virus vaccines against EV-A71 are commercially available in Mainland China. Furthermore, the mechanisms underlying the infectivity and pathogenesis of EV-A71 remain to be better understood. By adaptation of an EV-A71 B5 strain in monkey Vero cells in the presence of brilliant black BN (E151), an anti-EV-A71 agent, a double mutant with VP1-V238A,K244R emerged whose infection was enhanced by E151. The growth of the reverse genetics (RG) mutant RG/B5-VP1-V238A,K244R (RG/B5-AR) was promoted by E151 in Vero cells but inhibited in other human and murine cells, while its parental wild type, RG/B5-wt, was strongly prevented by E151 from infection in all tested cells. In the absence of E151, RG/B5-AR exhibited defective cell entry/exit, resulting in reduced viral transmission and growth *in vitro*. It had augmented binding affinity to sulfated glycans, cells, and tissue/organs, which probably functioned as decoys to restrict viral dissemination and infection. RG/B5-AR was also attenuated, with a 355 times higher 50% lethal dose (LD_50_) and a shorter timing of virus clearance than those of RG/B5-wt in suckling AG129 mice. However, it remained highly immunogenic in adult AG129 mice and protected their suckling mice from lethal EV-A71 challenges through maternal neutralizing antibodies. Overall, discovery of the attenuated mutant RG/B5-AR contributes to better understanding of virulence determinants of EV-A71 and to further development of novel vaccines against EV-A71.

**IMPORTANCE** Enterovirus A71 (EV-A71) is highly contagious in children and has been responsible for thousands of deaths in Asia-Pacific region since the 1990s. Unfortunately, the virulence determinants and pathogenesis of EV-A71 are not fully clear. We discovered that a novel EV-A71 mutant, VP1-V238A,K244R, showed growth attenuation with reduced efficiency of cell entry/exit. In the Vero cell line, which has been approved for manufacturing EV-A71 vaccines, the growth defects of the mutant were compensated by a food dye, brilliant black BN. The mutant also showed augmented binding affinity to sulfated glycans and other cellular components, which probably restricted viral infection and dissemination. Therefore, it was virulence attenuated in a mouse model but still retained its immunogenicity. Our findings suggest the mutant as a promising vaccine candidate against EV-A71 infection.

## INTRODUCTION

Enterovirus A71 (EV-A71) is classified in the Enterovirus A species in the Enterovirus genus of the Picornaviridae family, which contains many human pathogens. EV-A71 is a nonenveloped, icosahedral particle with a positive-sense, single-stranded RNA genome. Its capsid is about 30 nm in diameter and consists of 60 copies of protomers, each of which has four structural proteins, namely, VP1, VP2, VP3, and VP4 (1–2). VP1 is the most immunogenic protein to elicit neutralizing antibodies and is involved in cell entry. Based on the nucleotide sequence of VP1, EV-A71 strains are divided into seven distinct genogroups, namely, A, B, C, D, E, F, and G. Moreover, genogroups B and C are further divided into subgenogroups B1 to B5 and C1 to C5, respectively (3–5). Although EV-A71 infection usually causes mild symptoms, including hand, foot, and mouth disease (HFMD), it occasionally leads to neurological complications, which can be lethal (6–10). Outbreaks of EV-A71 infection among children in the Asia-Pacific region have resulted in several thousand deaths during the last 30 years (11).

Three types of formalin-inactivated whole-virus EV-A71 vaccines have been commercially available in Mainland China since 2016 (12), but no EV-A71 vaccine has approved in other regions to date. All of these vaccines use EV-A71 subgenogroup C4 wild-type strains as virus seeds which grow in cultured African green monkey kidney Vero or human diploid KMB-17 cells (13–15). Therefore, their safety control is a challenge because specialized vaccine manufacturing equipment with biosafety level 2+ is required, and incomplete inactivation may lead to vaccine-derived infections. Although the inactivated EV-A71 vaccines have an advantage over live attenuated vaccines for safety reasons, they require at least two doses to elicit a solid humoral immunity against EV-A71 infection, and the immunity wanes after the first 6 months of vaccination (16). On the other hand, live attenuated EV-A71 vaccines could elicit both humoral and cell-mediated immunities to provide strong and durable protection based on the success of attenuated poliovirus vaccines, but this strategy needs to overcome safety issues about virulence determinants and pathogenesis of EV-A71. Identification of attenuated EV-A71 mutants not only unveils the mechanisms underlying viral infection and pathogenesis but also benefits the development of attenuated EV-A71 vaccines. For example, a temperature-sensitive mutant of the EV-A71 BrCr strain exhibits attenuation in cynomolgus monkeys, induces high levels of neutralizing antibodies with cross-reactivity to other EV-A71 genogroups, and protects the monkeys against the lethal challenge of EV-A71 (17, 18). A high-fidelity double mutant of EV-A71 with L123F and G64R in the viral polymerase 3D is also attenuated in immunodeficient AG129 mice (19). Moreover, combination of different attenuation mutations in one strain could be synergic and reduce virulence reversion (20, 21).

Efficient infection of EV-A71 relies on the success of every stage of its life cycle. At first, EV-A71 attaches to the surface of host cells by interacting with viral attachment factors, which includes scavenger receptor B2 (SCARB2) (22), P-selectin glycoprotein ligand-1 (PSGL1) (23), and heparan sulfate (HS), a type of sulfated glycosaminoglycan (GAG) (24). After viral internalization through endocytosis, EV-A71 undergoes uncoating, which is induced by low pH and the uncoating factors SCARB2 and cyclophilin A (CypA) (25), and finally releases viral genome into the cytosol. Biosynthesis of viral proteins and genomes happens in the cytosol and is followed by virus assembly and progeny release. Previous studies indicate that strong binding affinity of viruses, including that of EV-A71, to sulfated GAGs enhances viral attachment and infection *in vitro* but restricts viral spread/dissemination and results in virulence attenuation *in vivo* (26–27). The positively charged vertex of the 5-fold axis of EV-A71, mainly determined by amino acids at VP1-98, VP1-145, VP1-242, and VP1-244, is involved in the viral attachment to HS (28, 29) and affects viral pathogenesis (26, 27, 30, 31) and immunogenicity (32, 33).

Brilliant black BN, a food azo dye with the E number E151, has been identified previously as a promising drug candidate against almost all circulating EV-A71 strains (34). By adaptation of an EV-A71 B5 strain in the presence of E151 in a vaccine production Vero cell line, the infection of the 14th passaged mutant was found to be partially dependent on E151. In contrast to the parental wild type, RG/B5-wt, which was inhibited by E151, the reverse genetics (RG) mutant RG/B5-AR with VP1-V238A,K244R produced more viral proteins, RNAs, and progeny in the presence of E151 in Vero cells. However, both RG/B5-wt and RG/B5-AR were still E151 sensitive in tested human and murine cells. On the other hand, in the absence of E151, RG/B5-AR exhibited growth attenuation with defective cell entry/exit. It had augmented binding affinity to sulfated glycans, cells, and tissue/organs, which might trap the virions to reduce the viral dissemination and infection. Moreover, RG/B5-AR was also attenuated in 5-day-old immunodeficient AG129 mice, and its 50% lethal dose (LD_50_) was 355 times higher than that of RG/B5-wt. However, it was highly immunogenic in adult AG129 mice to elicit EV-A71 specific neutralizing antibodies and protect their suckling mice from lethal EV-A71 challenges. The properties of RG/B5-AR contribute to the understanding of EV-A71 virulence and suggest a novel way to develop live attenuated EV-A71 vaccines.

## RESULTS

### E151 enhanced infection of Vero/B5-E151-P14 in Vero cells.

**FIG 1:**
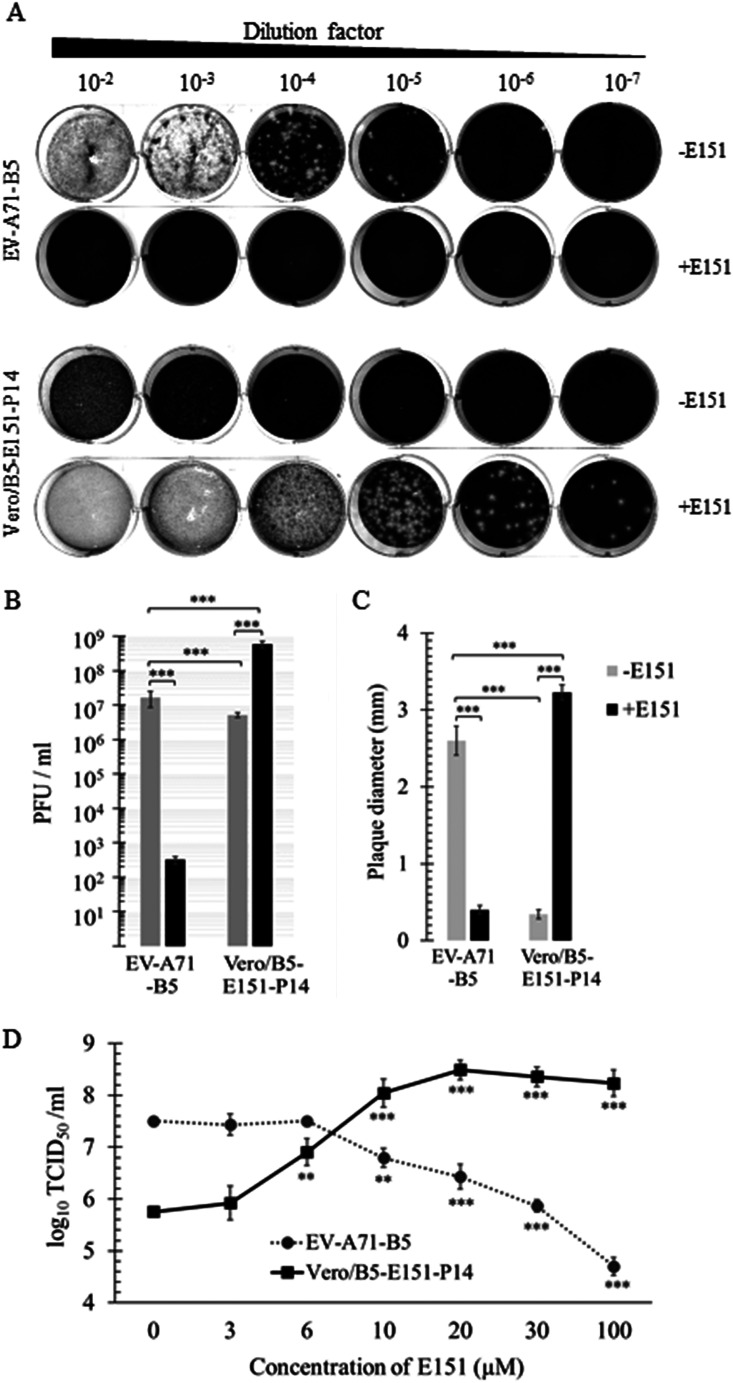
Selection and characterization of Vero/B5-E151-P14. EV-A71-B5 was passaged in Vero cells in the presence of E151. Growth of the 14th-passage virus Vero/B5-E151-P14 was enhanced by E151. Plaque morphology (A), PFU (B), and plaque diameter (C) of EV-A71-B5 and Vero/B5-E151-P14 in the presence or absence of 100 μM E151 (+E151 or −E151). A representative image of triplicate independent experiments for each condition is shown in panel A. The data in panels B and C are shown as the mean values ± standard errors (means ± SEM) of three independent experiments (*n* = 6 in panel B and *n* = 30 in panel C; two-tailed unpaired Student’s *t* test). (D) E151-concentration dependent growth of Vero/B5-E151-P14 in Vero cells. Cells (2 × 10^5^) were infected with EV-A71-B5 or Vero/B5-E151-P14 at a multiplicity of infection (MOI) of 0.1 for 24 h in the presence of E151, whose concentration ranged from 0 to 100 μM. The virus progeny of EV-A71-B5 and Vero/B5-E151-P14 were titrated in Vero cells in Dulbecco’s modified Eagle’s medium supplemented with 10% fetal bovine serum (DMEM-10) and DMEM-10 containing 100 μM E151, respectively. Lines indicate the means ± SEM (*n* = 3, one-way analysis of variance [ANOVA] with Dunnett’s posttest compared to those at 0 μM E151 for each virus).

The food dye brilliant black BN (E151) has been previously identified to inhibit infection of EV-A71 and coxsackievirus A16 (CVA-16) (34). In this study, adaptation of EV-A71 isolates in Vero cells in the presence of E151 led to mutants whose growth was enhanced by E151. A clinical isolate, EV-A71-B5, for example, produced about 1.67 × 10^7^ PFU/ml with a mean plaque diameter of 2.6 mm in the absence of E151, while 100 μM E151 reduced the mean plaque number and diameter to 317 PFU/ml and 0.34 mm, respectively (Fig. 1A to C). In contrast to the parental EV-A71-B5, passages of EV-A71-B5 gradually produced fewer and smaller plaques in the absence of E151, but more and larger plaques in the presence of E151 during serial blind passage (data not shown). The 14th passage of EV-A71-B5 in Vero cells in the presence of E151, named Vero/B5-E151-P14, exhibited growth defects with decreased plaque number (5.17 × 10^6^ PFU/ml) and diameter (0.40 mm) in the absence of E151. However, its infection was compensated and became more efficient with increased plaque number (5.83 × 10^8^ PFU/ml) and diameter (3.23 mm) in the presence of 100 μM E151 (Fig. 1A to C). The inhibition of EV-A71-B5 and enhancement of Vero/B5-E151-P14 by E151 were dose dependent. The growth titers of Vero/B5-E151-P14 were significantly promoted by E151 at concentrations of ≥6 μM and reached the highest level at 20 μM. Higher concentrations of E151 did not further increase viral growth (Fig. 1D).

### VP1-V238A,K244R is responsible for the E151-enhanced infection of Vero/B5-E151-P14.

**FIG 2:**
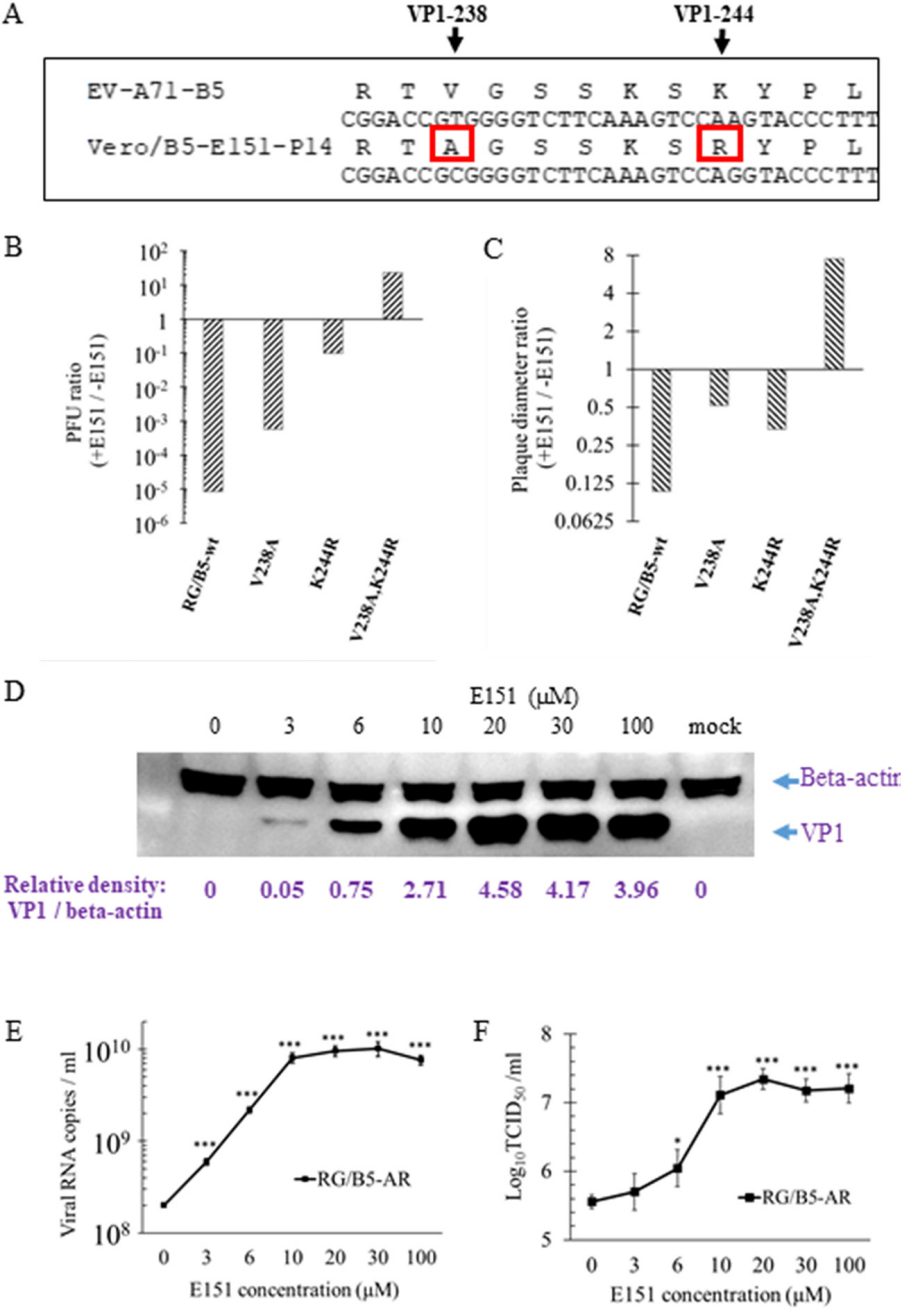
Identification of mutations responsible for E151-enhanced infection of Vero/B5-E151-P14. (A) Comparison of the VP1 sequences of EV-A71-B5 and Vero/B5-E151-P14 indicated two amino acid mutations, V238A and K244R (highlighted by red rectangles). The ratios of average PFU (B) and average plaque diameter (C) of B5 variants in the presence of 100 μM E151 (+E151) to those in the absence of E151 (−E151) are presented in Table 2. (D to F) E151 concentration-dependent replication of RG/B5-V238A,K244R (RG/B5-AR). In total, 2 × 10^5^ Vero cells were infected at an MOI of 0.1 PFU/cell (based on the titer in the presence of 100 μM) for 24 h in 24-well plates in 500 μl of DMEM-10 containing different concentrations of E151. Its VP1 antigen (D), RNA copies (E), and virus progeny (F) were quantified by Western blotting, quantitative reverse transcription-PCR (qRT-PCR) and 50% tissue culture infective dose (TCID_50_) assay, respectively. A representative figure and the average relative density ratio of VP1 to beta-actin of the 3 independent experiments in panel D are shown. Lines in panels E and F indicate the means ± SEM (*n* = 3; one-way ANOVA with Dunnett’s posttest compared to those at 0 μM E151).

To identify the mutations determining the E151-enhanced infection of Vero/B5-E151-P14, its genome was sequenced and compared to that of the parental EV-A71-B5. The result indicated that two amino acid substitutions, VP1-V238A (substitution of A for V at VP1-238, GUG→GCG) and VP1-K244R (AAG→AGG), occurred at the binding site of E151 (Fig. 2A). The genomic sequences of less-passaged viruses showed that VP1-K244R and VP1-V238A emerged as early as passages P3 and P8, respectively (data not shown). The two mutations V238A (GUG→GCC) and K244R (AAG→CGC) with new genetic codes were introduced alone or together into an infectious clone of EV-A71-B5, named pJET-EV-A71-B5-wt (wild type), by site-directed mutagenesis. Each of the infectious clones was transfected into Vero cells in duplicates. After removal of the transfection remnants at 6 h posttransfection, one well of each transfected clone was cultured in Dulbecco’s modified Eagle’s medium (DMEM) supplemented with 10% fetal bovine serum (FBS) (DMEM-10) and the other in DMEM-10 with 100 μM E151 to rescue the RG viruses. After 3 days, the transfected cells were subjected three times to freeze-thawing, and the viruses in supernatant were inoculated into fresh Vero cells in the presence or absence of E151. Based on the cytopathic effects (CPE) induced by virus infection, RG/B5-wt, RG/B5-V238A, and RG/B5-K244R grew better in the absence of E151, while growth of the double mutant RG/B5-AR with VP1-V238A,K244R was enhanced in the presence of E151 (Table 1).

**TABLE 1:** Severity of CPE of Vero cells transfected or infected by EV-A71-B5 RG variants in the presence or absence of 100 μM E151^*a*^

RG variant	CPE^*b*^						Effect of E151 on CPE
	Transfection		Passage 1		Passage 2		
	−E151	+E151	−E151	+E151	−E151	+E151	
Wild type (wt)	+/−	−	++	−	+++	−	Inhibition
V238A	−	−	+	−	++	−	Inhibition
K244R	−	−	+	+/−	++	+/−	Slight inhibition
V238A,K244R (AR)	−	+/−	+/−	++	+	+++	Enhancement

a Vero cells were transfected with reverse genetics (RG) viral infectious clones or infected with RG viruses in the presence or absence of E151. The effect of E151 on the cytopathic effect (CPE) induced by viral infection was observed and recorded daily up to 72 h.

b +++, CPE in >50% of cells at 24 h postinfection or posttransfection; ++, CPE in >50% of cells at 48 h postinfection or posttransfection; +. CPE in >50% of cells at 72 h postinfection or posttransfection; +/−, CPE in a few cells at 72 h postinfection or posttransfection; −, no CPE.

The second-passage viruses RG/B5-wt, RG/B5-V238A, and RG/B5-K244R propagated without E151, and RG/B5-AR propagated with E151 were harvested after >80% infected cells showed CPE and titer was further determined by plaque assay in Vero cells in the presence or absence of 100 μM E151 (Table 2). The results showed that RG/B5-wt, similarly to its clinical isolate, was highly sensitive to E151, with a PFU ratio (+E151/−E151, defined as the PFU in the presence of E151 divided by the PFU in the absence of E151) of ∼10^−5^, while the single mutants RG/B5-V238A and RG/B5-K244R were less sensitive to E151, with PFU ratios of 5 × 10^−3^ and 0.1, respectively (Fig. 2B), suggesting that amino acids at VP1-238 and VP1-244 determine viral sensitivity to E151 in Vero cells. RG/B5-AR, however, was enhanced by E151,as its PFU ratio reached ∼23, which is greater than 1 (Fig. 2B). The virus plaque size results also indicated that the single mutations V238A and K244R each reduced viral sensitivity to E151, with higher plaque diameter ratios (the mean plaque diameter in +E151 divided by the mean plaque diameter in −E151) than that of RG/B5-wt, but the ratios were still less than 1. In contrast, the double mutation V238A,K244R converted EV-A71-B5 to be E151 enhanced, as its plaque diameter ratio was about 8 (Table 2 and Fig. 2C). Similarly to that of Vero/B5-E151-P14, the growth of RG/B5-AR was enhanced by E151 in a concentration-dependent manner. After 24 h of infection in Vero cells at a multiplicity of infection (MOI) of 0.1 PFU/cell (based on the titer in the presence of 100 μM E151), VP1 antigen (Fig. 2D), RNA copies (Fig. 2E), and progeny titers (Fig. 2F) of RG/B5-AR rose incrementally with the increased E151 concentrations and reached the highest level at 20 μM. In human rhabdomyosarcoma (RD) cells, however, all three mutants were still strongly inhibited by E151 and failed to generate visible plaques in the presence of 100 μM E151 (Table 2).

**TABLE 2:** Plaque assay of EV-A71-B5 RG variants (second passage) in Vero and RD cells in the presence or absence of 100 μM E151

RG variant	Plaque assay results for:							
	Vero cells				RD cells			
	−E151		+E151		−E151		+E151	
	PFU/ml^*a*^	Plaque diam (mm)^*b*^	PFU/ml	Plaque diam (mm)	PFU/ml	Plaque diam (mm)	PFU/ml	Plaque diam (mm)^*c*^
Wild type (wt)	3 ± 0.82 × 10^7^	2.58 ± 0.12	2.50 ± 0.58 × 10^2^	0.28 ± 0.05	4.75 ± 0.96 × 10^6^	1.50 ± 0.07	0	NA
V238A	0.48 ± 0.10 × 10^7^	2.20 ± 0.09	2.75 ± 0.96 × 10^3^	1.14 ± 0.07	0.43 ± 0.15 × 10^6^	0.83 ± 0.06	0	NA
K244R	1.23 ± 0.52 × 10^7^	1.00 ± 0.07	1.20 ± 0.54 × 10^6^	0.34 ± 0.06	2.25 ± 0.96 × 10^6^	0.41 ± 0.05	0	NA
V238A,K244R (AR)	0.65 ± 0.10 × 10^7^	0.43 ± 0.06	1.50 ± 0.56 × 10^8^	3.23 ± 0.1	0.63 ± 0.13 × 10^6^	0.34 ± 0.06	0	NA

a Mean ± SEM of two independent experiments (*n* = 6) for each virus.

b Mean ± SEM of randomly selected plaques (*n* = 20) for each virus.

c NA, not applicable.

**FIG 3:**
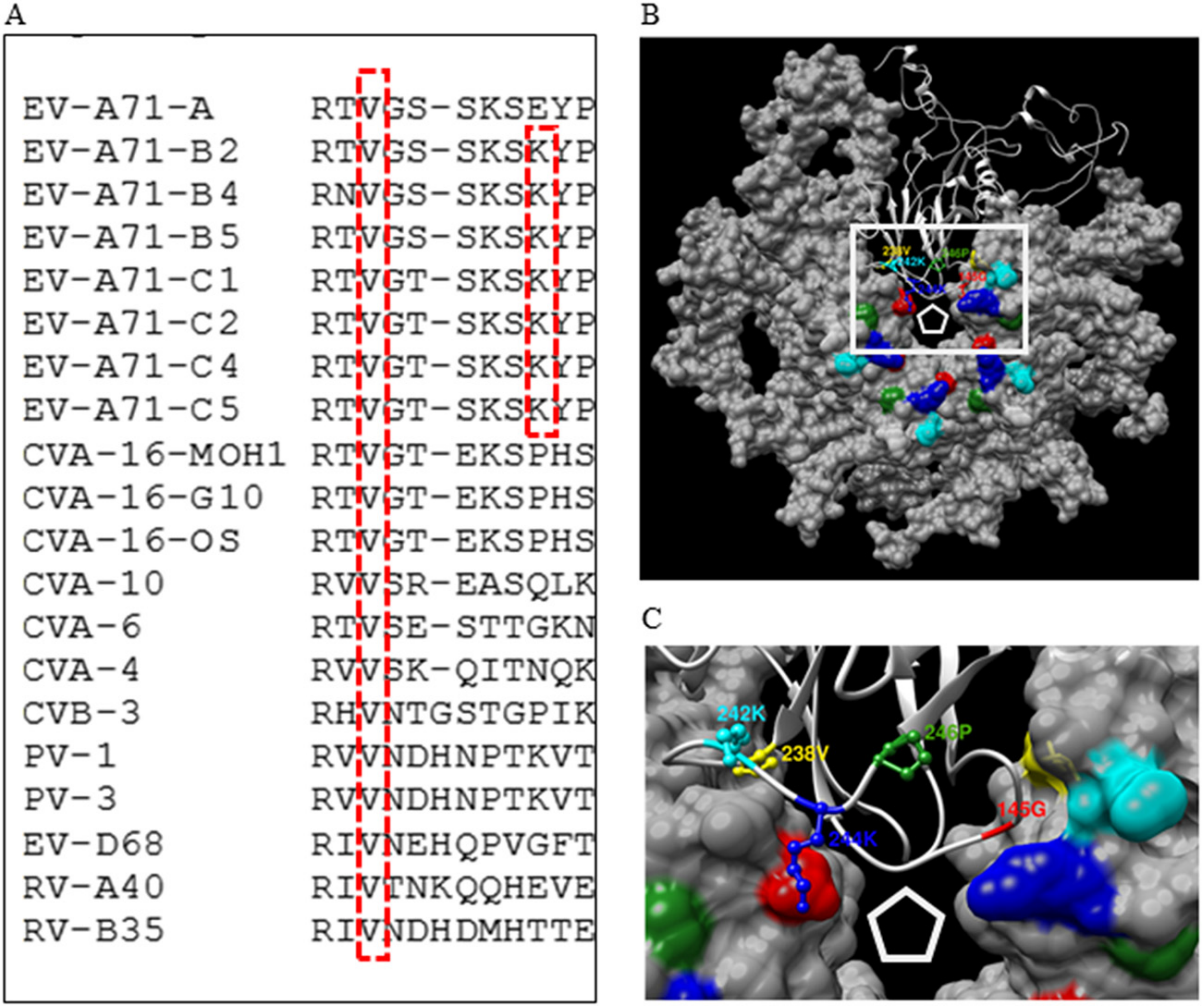
Conserved VP1-238A and VP1-244K in EV-A71. (A) Multiple-protein alignment of VP1s of enteroviruses. The VP1 proteins of 8 EV-A71 strains (EV-A71-A, EV-A71-B2, EV-A71-B4, EV-A71-B5, EV-A71-C1, EV-A71-C2, EV-A71-C4, and EV-A71-C5) and 3 CVA16 strains (CVA-16-MOH1, CVA-16-G10, and CVA-16-OS) were previously sequenced (34). The VP1 sequences of CVA-10 (GenBank accession number ARU09507), CVA-6 (accession number AUF49646), CVA-4 (accession number ATP75726), CVB-3 (accession number AIZ97146), PV-1 (accession number CAA24461, poliovirus type 1), PV-3 (accession number AAN85444, poliovirus type 3), EV-D68 (accession number ALJ53630, enterovirus D68), RV-A40 (accession number AFK65738, rhinovirus A40), and RV-B35 (accession number AFK65739, rhinovirus B35) were retrieved from the NCBI database. All sequences were aligned using Clustal W. (B) Three-dimensional structure of a VP1 pentamer of an EV-A71 virion (PDB ID 3VBS). The solid surface of four VP1 proteins and the rounded ribbon of one VP1 protein are presented. A white pentagon indicates the vertex of the viral 5-fold axis. Amino acids at VP1-145 (red), VP1-238 (yellow), VP1-242 (cyan), VP1-244 (blue), and VP1-246 (green), which modulate the interactions among E151, EV-A71, and host cells, are highlighted. (C) An enlarged view of a white rectangle in panel B. Highly conserved VP1-238V, VP1-242K, VP1-244K, and VP1-246P in the ball and stick form are all located at the HI loop near the vertex of the viral 5-fold axis.

The multiple-protein alignment of VP1 proteins of enteroviruses indicated that VP1-238V is highly conserved in human enteroviruses, including poliovirus and rhinovirus, while VP1-244K is only conserved in EV-A71 (Fig. 3A). Based on the crystal structure of an EV-A71 isolate (PDB identifier [ID] 3VBS), VP1-238V, VP1-242K, VP1-244K, and VP1-246P, which determine the sensitivity of EV-A71 to E151, are all located at the HI loop near the vertex of the viral 5-fold axis (Fig. 3B and C).

### *In vitro* growth attenuation of RG/B5-AR in the absence of E151.

**FIG 4:**
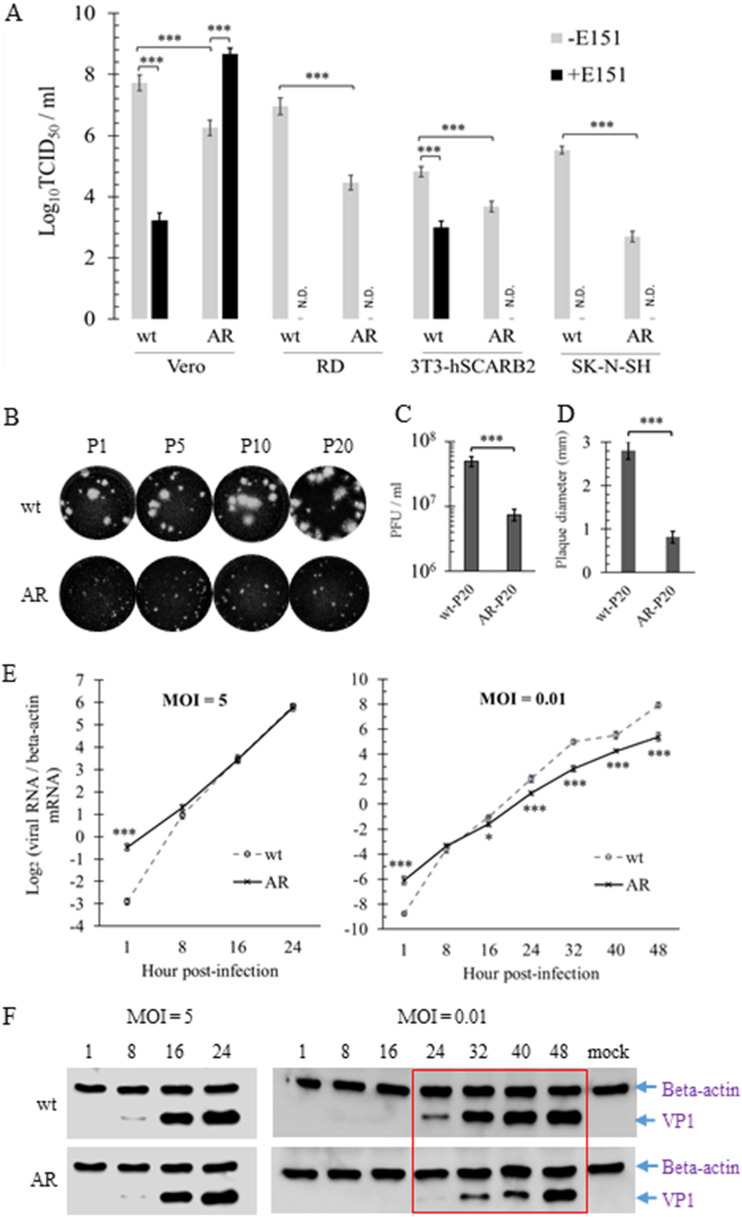
*In vitro* attenuation of RG/B5-AR in the absence of E151. (A) In the absence of E151, the titers of RG/B5-AR (AR) was lower than those of RG/B5-wt (wt). The passage 2 (P2) viruses wt and AR were titrated in Vero, RD, 3T3-hSCARB2, and SK-N-SH cells in the presence (+) or absence (−) of 100 μM E151. The TCID_50_ was calculated based on viral antigen positivity by immunofluorescent assay (IFA) in SK-N-SH and cytopathic effect (CPE) in the other three cell lines at 3 days postinfection in three independent experiments (*n* = 3; two-tailed unpaired Student’s *t* test; N.D., not detectable). (B to D) Attenuation of AR was stable. Plaque morphologies (B) of different passages (P1, P5, P10, and P20) of wt and AR in Vero cells. PFU (D) and plaque diameter (E) of the twentieth passage wt-P20 and AR-P20 are presented as the means ± SEM of three independent experiments (*n* = 6 in panel D and *n* = 30 in panel E; two-tailed unpaired Student’s *t* test). (E and F) One-step growth kinetics demonstrated the growth attenuation of AR at a low but not a high MOI. Vero cells were infected by wt or AR at an MOI of 5 or 0.01. Viral RNA (E) and VP1 protein (F) were quantified at different hours postinfection by qRT-PCR and Western blot analysis, respectively. Lines in panel E indicate the means ± SEM (*n* = 6; two-tailed unpaired Student’s *t* test). A red rectangle in panel F shows that the VP1 protein expression of AR was less than that of the wt from 24 to 40 h postinfection when the MOI was 0.01.

In the absence of E151, all of the mutants RG/B5-V238A, RG/B5-K244R, and RG/B5-AR exhibited growth attenuation with fewer and smaller plaques in both Vero and RD cells compared to those of RG/B5-wt (Table 2). RG/B5-wt and RG/B5-AR were further titrated in Vero, RD, mouse 3T3 expressing human SCARB2 (3T3-hSCARB2), and human SK-N-SH neuroblastoma cells using 50% tissue culture infective dose (TCID_50_) assay. Like the results of the plaque assay, the titer of RG/B5-AR was much lower than that of RG/B5-wt in all four cell lines. Moreover, the lower titer of RG/B5-AR was compensated by E151 only in Vero cells, and was further reduced in other three cell lines (Fig. 4A). Although serial passage of RG/B5-AR in the absence of E151 in Vero cells (P1 to P20) slightly increased its growth, it always exhibited lower infectivity than that of RG/B5-wt (Fig. 4B). For example, the plaque assay of the twentieth passages of RG/B5-wt and RG/B5-AR, named wt-P20 and AR-P20, respectively, indicated that AR-P20 produced fewer and smaller plaques than wt-P20 in Vero cells. The mean growth titer and plaque diameter of wt-P20 were 5 × 10^7^ PFU/ml and 2.81 mm, respectively, while those of AR-P20 were 7.5 × 10^6^ PFU/ml and 0.82 mm, respectively (Fig. 4C and D). The results demonstrated that attenuation of RG/B5-AR was stable after the passage in the absence of E151, implying the potential use of RG/B5-AR as a vaccine candidate, provided that stability will also be confirmed after passage *in vivo*.

To understand the mechanism underlying the attenuation of RG/B5-AR, one-step growth kinetics of RG/B5-wt and RG/B5-AR in Vero cells at an MOI of 5 or 0.01 were compared. At the high MOI of 5, both of the viruses produced indistinguishable amount of viral RNA and VP1 protein from 8 to 24 h postinfection. In contrast, at the low MOI of 0.01, RG/B5-AR generated lower levels of viral RNA and VP1 protein than did RG/B5-wt from 16 to 48 h postinfection (Fig. 4E and F). The results indicated that the RG/B5-AR had the same replication rate but a lower transmission rate than RG/B5-wt *in vitro*.

### Reduced cell entry and exit efficiency of RG/B5-AR.

**FIG 5:**
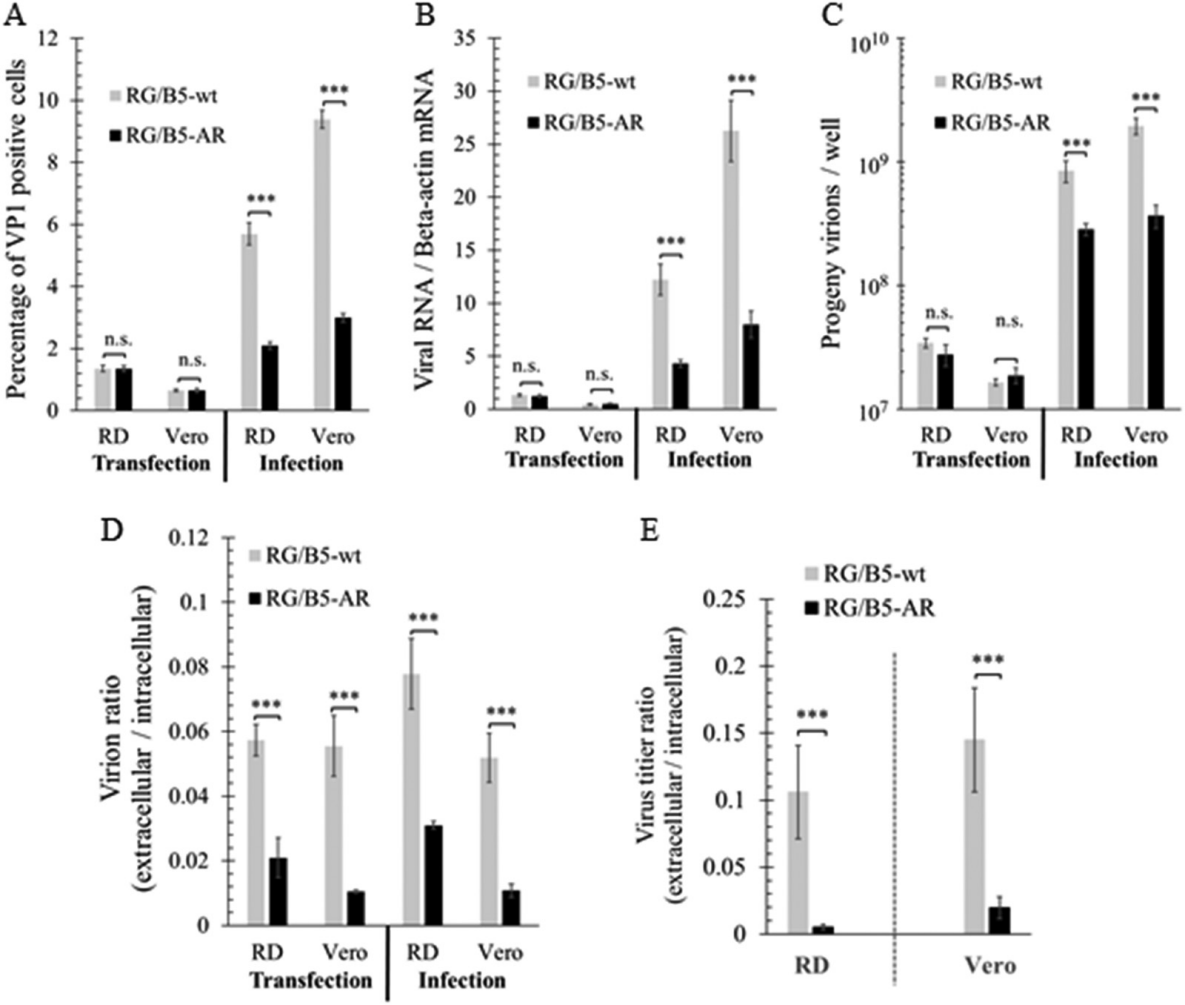
Reduced cell entry and exit of RG/B5-AR. (A to D) RG/B5-AR had less efficient cell entry and exit than RG/B5-wt in cell entry bypass assay. RD and Vero cells were transfected with purified viral genomic RNA at 200 copies/cell or infected with viruses at 200 virions/cell. At 10 h posttransfection or infection, the percentage of VP1-positive cells, relative viral RNA level, and number of intracellular and extracellular progeny virions were quantified by IFA or qRT-PCR for two independent experiments (*n* = 6; two-tailed unpaired Student’s *t* test; n.s., not significant). (E) Virus progeny release of RG/B5-AR was less efficient than that of RG/B5-wt. Cells were infected with RG/B5-wt or RG/B5-AR at an MOI of 0.01 for 48 h. Extracellular and intracellular virus progeny were titrated in Vero cells and their ratios were calculated for two independent experiments as the means ± SEM (*n* = 6; two-tailed unpaired Student’s *t* test).

Cell entry bypass assay was performed by transfecting genomic RNAs of RG/B5-wt or RG/B5-AR into RD and Vero cells at 200 copies/cell. At 10 h posttransfection, the percentage of VP1 antigen-positive cells, level of viral RNA (the ratio relative to beta-actin mRNA), and progeny virions were quantified and found not to be significantly different between the two genomic RNAs (Fig. 5A to C), implying that RG/B5-AR and RG/B5-wt possessed the same efficiencies in viral protein expression, genome replication, and virus assembly inside the two cell lines. In contrast, RG/B5-AR generated a smaller percentage of VP1-positive cells and lower levels of viral RNA and progeny virions than RG/B5-wt during the virus infection assay, in which the cells were infected by RG/B5-AR or RG/B5-wt at 200 virions/cell for 10 h (Fig. 5A to C). Therefore, the cell entry efficiency of RG/B5-AR was lower than that of RG/B5-wt in both RD and Vero cells.

In order to evaluate the efficiency of virus exit or progeny release *in vitro*, the ratios of extracellular to intracellular progeny virions of RG/B5-wt and RG/B5-AR were also quantified and compared by quantitative reverse transcription-PCR (qRT-PCR) in the two assays described above. RG/B5-AR had much lower ratios than RG/B5-wt in both assays (Fig. 5D), suggesting that progeny release of RG/B5-AR was defective. In another experiment, RD and Vero cells were infected at an MOI of 0.01 PFU/cell. When all cells exhibited a CPE at 48 h postinfection, titer of the extracellular and intracellular virus progeny was determined by TCID_50_ assay. For RG/B5-wt, the titer ratios of extracellular virus to intracellular virus were about 0.106 and 0.145 in RD and Vero cells, respectively. For RG/B5-AR, the titer ratios declined significantly to 0.005 and 0.020 in RD and Vero cells, respectively (Fig. 5E). Therefore, *in vitro*, the viral exit of RG/B5-AR was less efficient than that of RG/B5-wt.

### Changed binding affinity of RG/B5-AR to viral attachment and uncoating factors.

**FIG 6:**
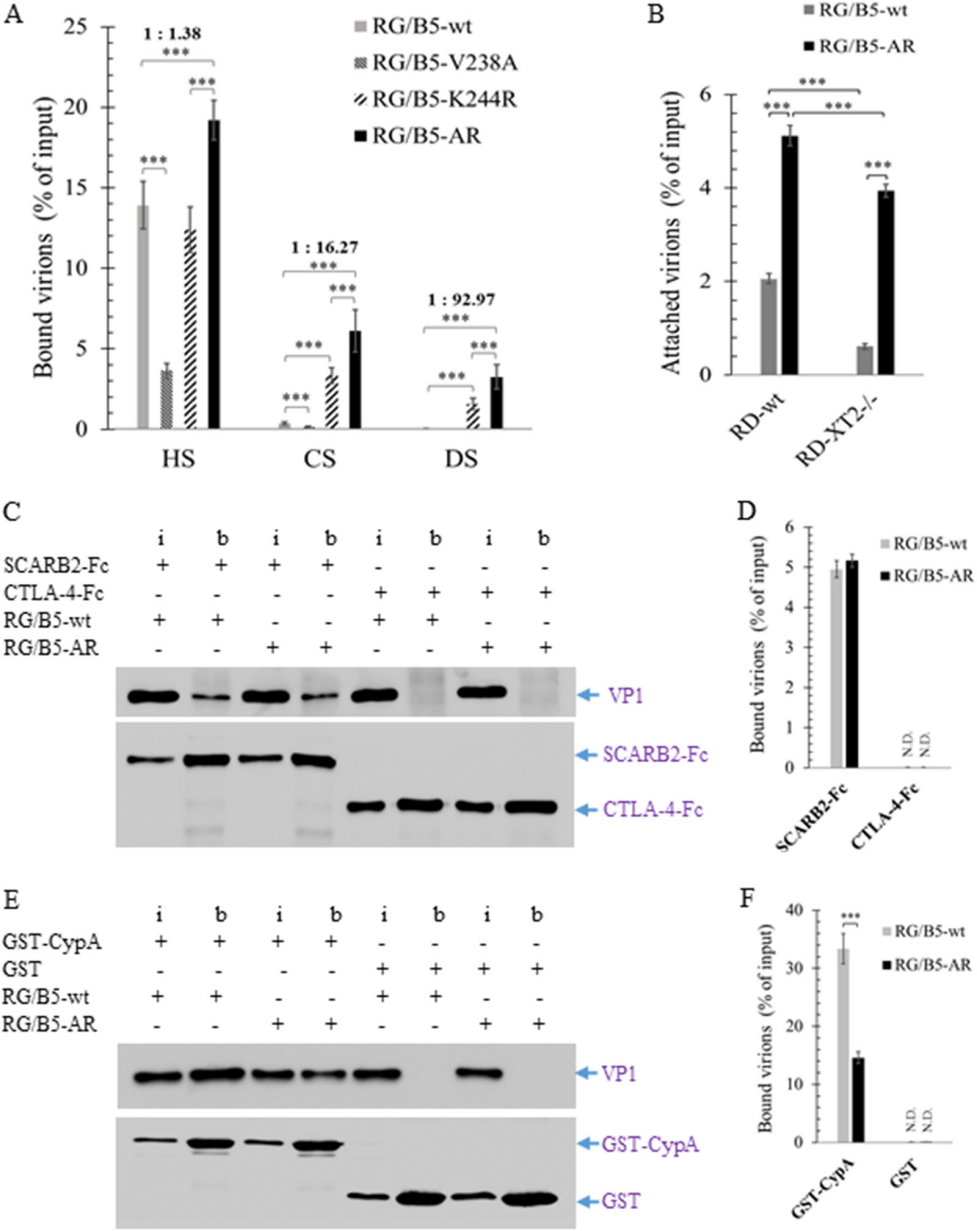
Changed binding affinity of RG/B5-AR to viral attachment and uncoating factors. (A) RG/B5-AR had stronger binding affinity than RG/B5-wt with sulfated GAGs. Purified RG/B5-wt, RG/B5-V238A, RG/B5-K244R, or RG/B5-AR was incubated with heparan sulfate (HS)-, chondroitin sulfate (CS)-, or dextran sulfate (DS)-conjugated agarose resin. the percentages of input virions bound to sulfated GAGs were quantified by qRT-PCR for two independent experiments and are presented as the means ± SEM (*n* = 4; two-tailed unpaired Student’s *t* test). Ratios of bound RG/B5-wt to bound RG/B5-are were also shown above the brackets. (B) RG/B5-AR had higher binding affinity than RG/B5-wt with RD and RD-XT2^−/−^. Ice-cold virions and EDTA-detached cells were incubated at 4°C, and cell-attached virions were quantified by qRT-PCR for two independent experiments (*n* = 6; two-tailed unpaired Student’s *t* test). (C and D) Binding affinity of RG/B5-AR with human SCARB2 remained the same as that of RG/B5-wt. (E and F) Binding affinity of RG/B5-AR with human CypA was lower than that of RG/B5-wt. Virions were incubated with recombinant protein SCARB2-Fc, CTLA-4-Fc, glutathione *S*-transferase (GST)-CypA, or GST. The proteins interacting virions with were pulled down by protein A/G or glutathione Sepharose beads and quantified by Western blotting. (C and E) Representative images of two independent experiments under each condition using ImageLab are presented (i, 20% of input; b, bound; +, addition; −, no addition) and the percentages of bound virions are presented as the means ± SEM (*n* = 4; two-tailed unpaired Student’s *t* test; N.D., not detectable).

As RG/B5-AR had defective cell entry/exit, its binding affinity to viral attachment and uncoating factors, including HS, SCARB2, and CypA, was further evaluated. Compared to RG/B5-wt, the binding affinity of RG/B5-AR to HS-conjugated agarose resin increased 38% (Fig. 6A). Moreover, RG/B5-AR or single-mutant RG/B5-K244R strongly bound to chondroitin sulfate (CS; a ubiquitous sulfated GAG on the mammalian cell surface) and dextran sulfate (DS; a common bacterial sulfated glycan), which are not attachment factors of EV-A71 wild-type strains. The binding affinities of RG/B5-AR to CS and DS, in particular, were 16.27 and 92.97 times that of RG/B5-wt, respectively (Fig. 6A), implying that these sulfated glycans probably trapped RG/B5-AR to attenuate its infection. When the xylosyltransferase 2 (XT2) gene, responsible for biosynthesis of CS and HS, was knocked out of RD cells (RD-XT2^−/−^) (35), cell attachment of both RG/B5-wt and RG/B5-AR became weak. However, RG/B5-AR retained stronger attachment efficiency in RD-XT2^−/−^ cells than RG/B5-wt (Fig. 6B), suggesting that it had higher binding affinity to other components on cell surface, perhaps those with sulfation or other negatively charged groups.

In pulldown assays, RG/B5-AR and RG/B5-wt had the same binding affinity to recombinant protein SCARB2-Fc (Fig. 6C and D), while the binding affinity of RG/B5-AR to glutathione *S*-transferase (GST)-CypA dropped to 42% compared to that of RG/B5-wt (Fig. 6E and F). The results were consistent with the mutations V238A and K244R of VP1 being at the viral binding site of CypA, but not SCARB2 (25, 34). For comparison, none of the viruses interacted with negative-control CTLA-4-Fc or GST protein. As SCARB2 is the major receptor and uncoating factor of EV-A71 (22), the uncoating process of RG/B5-AR was not greatly affected by the weaker interaction with CypA in RD cells, which was corroborated by the observation that the growth kinetics of RG/B5-AR and RG/B5-wt were almost the same at the high MOI of 5 (Fig. 4E and F).

### Virulence attenuation of RG/B5-AR in AG129 mice.

**FIG 7:**
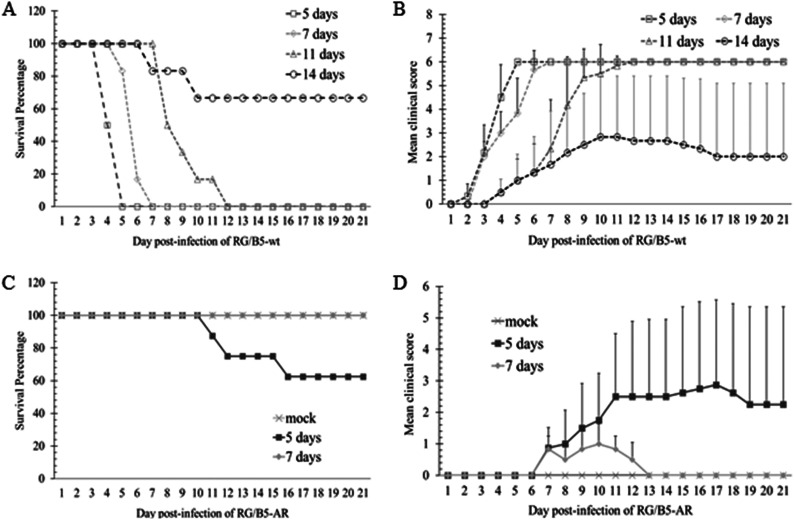
Age-dependent mortality of AG129 mice infected by RG/B5-wt or RG/B5-AR. AG129 mice (5, 7, 11, or 14 days old) were intraperitoneally infected with 10^8^ virions of RG/B5-wt (A and B) or RG/B5-AR (C and D). Control mice were administered phosphate-buffered saline (PBS) (mock). Mice were observed daily. Survival percentages (A and C) and clinical scores (B and D) were recorded up to 21 days postinfection.

**FIG 8:**
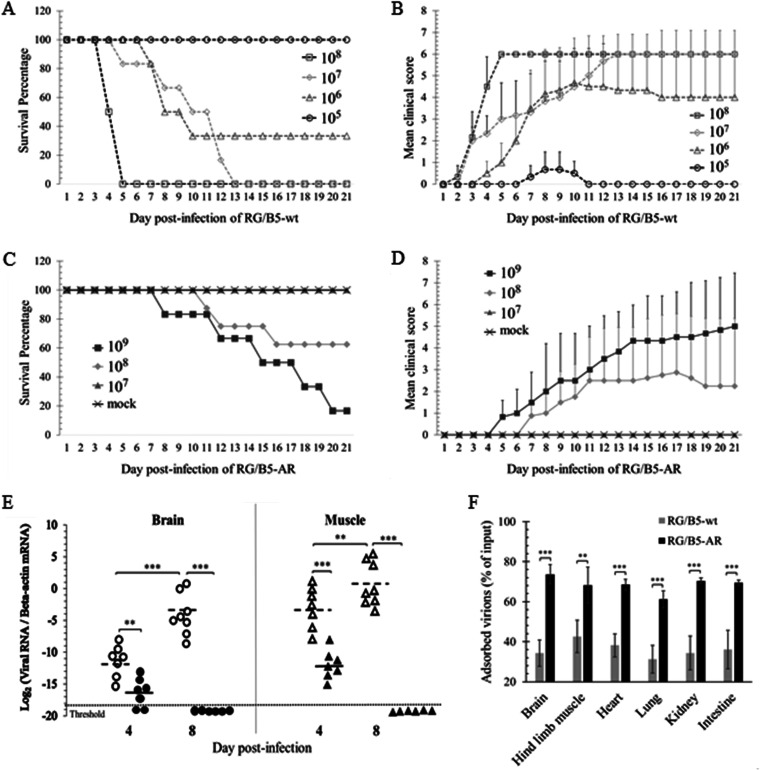
Attenuation of RG/B5-AR in 5-day-old AG129 mice. (A to D) RG/B5-AR was less virulent than RG/B5-wt. The 5-day-old AG129 mice were intraperitoneally injected with serially diluted doses of RG/B5-wt (A and B) or RG/B5-AR (C and D) ranging from 10^5^ to 10^9^ virions/mouse. Control mice were administered with PBS (mock). Survival percentages (A and C) and clinical scores (B and D) were recorded up to 21 days postinfection. (E) RG/B5-AR was less effective to replicate and/or disseminate than RG/B5-wt. Relative viral RNA levels in the brains and hind limb muscles of 5-day-old mice infected with 1.78 × 10^6^ virions of RG/B5-wt (unfilled circles and triangles) or RG/B5-AR (black filled circles and triangles) were quantified at 4 and 8 days postinfection. Black solid and dashed lines indicate means (*n* = 6 to 8; Mann-Whitney test). (F) RG/B5-AR was more easily adsorbed by mouse tissue/organs. After the input 10^6^ virions of RG/B5-wt or RG/B5-AR were incubated with 100 mg of insoluble fractions of six different tissue/organ homogenates in DMEM-10 at 4°C for 1 h, nonadsorbed virions in supernatants were quantified by plaque assay. Percentages of input virions adsorbed by tissue/organs are presented as the means ± SEM (*n* = 6; two-tailed unpaired Student’s *t* test).

As EV-A71 with increased binding affinity to sulfated GAGs are attenuated *in vivo* (26–28, 30, 31), the virulence of RG/B5-AR was further evaluated in AG129 mice. The mouse mortality caused by EV-A71 infection has been reported to be age dependent (36, 37). Therefore, groups of 5-, 7-, 11-, and 14-day-old AG129 mice were first infected intraperitoneally by 10^8^ virions of RG/B5-wt or RG/B5-AR, and their survival rates and clinical scores were recorded for 3 weeks. All of the 5-, 7-, and 11-day-old mice were killed by the parental wild type, RG/B5-wt, at 5, 7, and 12 days postinfection, respectively, and the 14-day-old mice were partially killed at 3 weeks postinfection (Fig. 7A and B). In contrast, the mutant RG/B5-AR only caused death in some of the 5-day-old mice and completely failed to induce severe symptoms, like limb paralysis, and death in the 7-day-old mice (Fig. 7C and D), implying that the virulence of RG/B5-AR was attenuated. Five-day-old mice were further used to evaluate the LD_50_ of RG/B5-wt and RG/B5-AR by intraperitoneal inoculation of virus diluents. After 3 weeks of infection, the LD_50_ of RG/B5-wt was determined to be 5.6 × 10^5^ virions, while the LD_50_ of RG/B5-AR was 2.0 × 10^8^ virions, which is about 355 times higher (Fig. 8A and C). Clinical scores also indicated that mice infected with RG/B5-AR exhibited milder clinical features at later time points than those infected with RG/B5-wt (Fig. 8B and D).

The attenuation of RG/B5-AR *in vivo* was further investigated by determining the viral RNA levels in the tissues and organs of infected mice. Five-day-old ARG129 mice were infected with 1.78 × 10^6^ virions of RG/B5-wt (∼3 LD_50_) or RG/B5-AR. The brains and hind limb muscles were harvested for total RNA extraction at 4 and 8 days postinfection, and the levels of viral RNA and beta-actin mRNA (internal control) were then quantified by qRT-PCR (Fig. 8E). At 4 days postinfection, a substantial number of copies of viral RNA was detected in the brains and hind limb muscles of both infected mice groups, but the viral RNA levels in the mice infected by RG/B5-AR were significantly lower than those in the mice infected by RG/B5-wt. At 8 days postinfection, the viral RNA levels in the brains and muscles of the RG/B5-wt infected mice increased, similarly to results reported in previous publications (36, 37), while the viral RNA levels in the brains and hind limb muscles of the RG/B5-AR-infected mice declined to undetectable levels, suggesting that RG/B5-AR replicated at a lower rate at the early stage of infection and was subsequently cleared.

EV-A71 and other viruses with higher binding affinity to sulfated GAGs (HS and/or CS) tend to be adsorbed and trapped by nonsusceptible cells and extracellular matrix in host tissue/organs. Therefore, adsorption of RG/B5-wt and RG/B5-AR to insoluble fractions of homogenized mouse brain, hind limb muscle, heart, lung, kidney, or intestine was further examined. After incubation of the 10^6^ virions and 100 mg of homogenates at 4°C for 1 h, the nonadsorbed virions in supernatants were quantified by plaque assay. As both viruses are stable during incubation (data not shown), reduction of the viral PFU is proportional to the number of adsorbed virions. The results showed that 30% to 40% of input RG/B5-wt and 60% to75% of input RG/B5-AR was adsorbed by the six different tissue/organ homogenates (Fig. 8F), suggesting that this higher adsorption of RG/B5-AR might reduce its dissemination and contribute to its virulence attenuation in mice.

### High immunogenicity of RG/B5-AR in AG129 mice.

**FIG 9:**
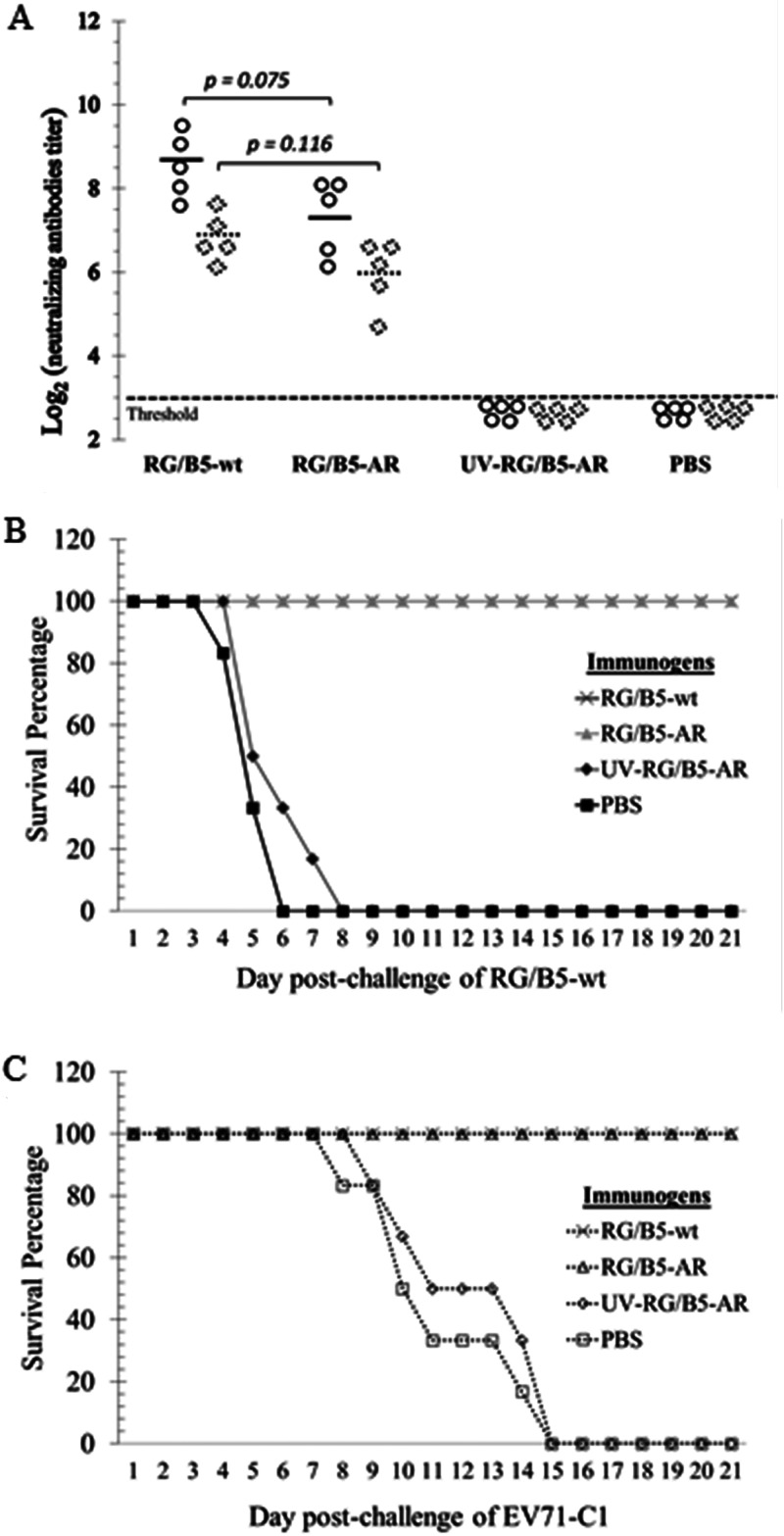
High immunogenicity of RG/B5-AR in AG129 mice. Adult females (*n* = 5) were immunized with purified RG/B5-wt, RG/B5-AR, UV-inactivated RG/B5-AR (UV-RG/B5-AR), or PBS, and then subjected to serum collection and breeding 4 weeks later. (A) RG/B5-AR elicited neutralizing antibodies equivalently to RG/B5-wt. Sera were tested in neutralizing antibody titer assay against homologous RG/B5-wt (circles) and heterologous EV-A71-C1 (dashed diamonds). Solid and dashed lines indicate means, and results are significant at *P* values of <0.05 (*n* = 5; Mann-Whitney test). (B and C) Immunization by RG/B5-AR in females protected their pups from lethal EV-A71 challenge. Pups (*n* = 6) of immunized females were intraperitoneally challenged with 100 50% lethal dose (LD_50_) of RG/B5-wt at 5 days old (B) or 10 LD_50_ of EV-A71-C1 at 14 days old (C). Survival percentages were recorded up to 21 days postchallenge.

The immunogenic potential of RG/B5-AR was evaluated in adult AG129 mice. Four female groups (*n* = 5) were immunized with 10^9^ virions of live RG/B5-wt, 10^9^ virions of live RG/B5-AR, 10^9^ virions of UV-inactivated RG/B5-AR (UV-RG/B5-AR), or phosphate-buffered saline (PBS). Sera collected at 4 weeks postimmunization indicated that one dose of RG/B5-AR induced strong EV-A71-specific neutralizing antibodies against both homologous RG/B5-wt and heterologous EV-A71-C1 strains. Although the mean of the neutralizing antibody titer (NAT) elicited by RG/B5-AR was lower than that of RG/B5-wt, their NATs were not significantly different. In contrast, UV-RG/B5-AR did not elicit detectable neutralizing antibodies, implying that viral replication is necessary to elicit a strong NAT in these immunized mice (Fig. 9A).

The immunized female mice were further used for breeding; their suckling mice were then challenged by 100 LD_50_ of RG/B5-wt at 5 days old or 10 LD_50_ of EV-A71-C1 at 14 days old. All pups from females immunized by RG/B5-AR or RG/B5-wt were completely protected from the lethal challenge through maternal antibodies and did not exhibit any symptoms, while all pups from the females immunized by UV-RG/B5-AR or PBS succumbed to the challenge (Fig. 9B and C). The survival percentages were concordant with the NAT results.

## DISCUSSION

EV-A71, as an RNA virus, has a high mutation rate and exists as a quasispecies (38–40). During *in vitro* adaptation, it can evolve into different cell-adapted mutants or populations with various phenotypes. Propagation of a clinical isolate, Tainan/5746/98, in Vero cells generates virulent progeny that kill transgenic mice with human SCARB2, while its passages in RD cells lose the virulence to kill the mice. The mutations E145G, V146I, and S241L of VP1 at the vertex of the viral 5-fold axis are found in the RD-adapted viruses and might be responsible for the virulence attenuation (41). In our previous studies, adaptation of EV-A71-B5 in RD cells in the presence of E151 leads to an E151-resistant mutant with E98K, G145E, and P246A in VP1 (35). In contrast, its adaptation in Vero cells in the presence of E151 resulted in a E151-enhanced mutant with V238A and K244R in VP1, as described in this study (Fig. 2). Interestingly, all mutations are also located at the vertex of the viral 5-fold axis, suggesting that this vertex is critical for infection by EV-A71 *in vitro*. It is well known that the vertex interacts with viral attachment factor HS and uncoating factor CypA in RD and Vero cells (24, 25). The VP1-V238A,K244R in RG/B5-AR greatly increased the viral binding affinity to HS (Fig. 6A), but it reduced the viral binding affinity to CypA (Fig. 6E and F). However, the mutations did not affect viral binding with the viral major receptor SCARB2, which interacts with the viral canyon region (Fig. 6C and D). Moreover, the vertex is also targeted by many anti-EV-A71 agents, including E151 (34). E151 retained interaction with RG/B5-AR, but it enhanced and inhibited viral infection in Vero and RD cells, respectively (Table 2). The distinct mutations between the RD- and Vero-adapted EV-A71 mutants indicate that the cellular components involved in the viral entry and infection differ between RD and Vero cells. This complicated virus-host interaction needs further investigation.

Efficiency in completion of each step of the viral life cycle is critical for EV-A71 to establish infection and cause disease in the hosts. In the absence of E151, RG/B5-AR exhibited growth attenuation with defective cell entry/exit in all tested cell lines. This attenuation was compensated by E151 in Vero cells but not in human and murine cells (Table 2 and Fig. 4A). Our preliminary data indicated that E151 only enhanced the cell entry of RG/B5-AR because the addition of E151 in the cell entry stage (0 to 3 h postinfection) but not in the virus intracellular replication stage (3 to 10 h postinfection) greatly increased the percentage of infected cells and the viral growth titer in Vero cells (data not shown). The mechanism underlying infection of RG/B5-AR being enhanced by E151 in Vero cells but inhibited by E151 in other cells will be studied in the future. Moreover, the attenuation of RG/B5-AR was unchanged after serial passages *in vitro* (Fig. 4B to D), implying that RG/B5-AR will be a good candidate for development of live attenuated EV-A71 vaccines.

The positively charged amino acids R and K are usually found in sulfated GAGs binding motifs (42, 43). VP1-242K and VP1-244K of EV-A71 are critical for the viral attachment to HS on the surface of host cells (28, 29). R has stronger electrostatic interactions with negatively charged molecules, such as sialic acids and sulfo groups, than K (43, 44). Substitution of R for K at VP1-244 enabled RG/B5-K244R to strongly bind to CS and DS, which did not interact with RG/B5-wt as attachment factors. Moreover, the combination of K244R and V238A in VP1 of RG/B5-AR further increased the viral binding affinity to all tested sulfated glycans, as well as to other cellular surface molecules (Fig. 6A and B). This augmented cell binding affinity of RG/B5-AR probably trapped it and harmed its cell entry/exit (Fig. 5), resulting in growth attenuation with reduced efficiency of viral transmission *in vitro* (Fig. 4).

Augmented cell binding of viruses sometimes restricts their dissemination and attenuates their virulence *in vivo*. Host cell adaptation of Japanese encephalitis virus (JEV), Murray Valley encephalitis virus (MVEV) (45), yellow fever virus (YFV) (46), and EV-A71 (29) significantly increases the viral binding affinity to sulfated GAGs, which are viral attachment factors that enhance the viral infection *in vitro*. However, a large amount of sulfated GAGs in the extracellular matrix can trap these cell-adapted viruses and prevent them from further infection *in vivo*. For example, substitution of the amino acid K or R for E at position 326 of the E protein of an attenuated YFV strain, 17D, gives the protein an increased net-positive charge to interact with sulfated GAGs, makes the virus more sensitive to heparin, and decreases the viral neuroinvasive ability in a SCID mouse model (46). For cell-adapted EV-A71 strains, substitution of G or Q for E at VP1-145 makes the vertex of the viral 5-fold axis more positively charged and increases the viral attachment affinity to HS and heparin (29). Similarly, EV-A71 strains with VP1-145G are less neurovirulent and fail to disseminate well in wild-type suckling mice (26, 30), transgenic mice expressing human SCARB2 (26), and cynomolgus monkeys (27, 31), compared to the strains with VP1-145E. In this study, the double mutation VP1-V238A,K244R in RG/B5-AR, which already has VP1-145G as RG/B5-wt, further augmented the viral binding affinity to sulfated glycans, cells, and tissue/organs (Fig. 6 and 8F). As expected, RG/B5-AR was less virulent than RG/B5-wt in suckling AG129 mice (Fig. 7 and 8).

Compared to RG/B5-wt, RG/B5-AR exhibited strong virulence attenuation, with an ∼355 times higher LD_50_ in 5-day-old AG129 mice (Fig. 8). Moreover, it was not lethal to older suckling mice, even at very high challenge doses (Fig. 7). The replication of RG/B5-AR was also found to decrease in the mice, with its RNA detectable in the brains and hind limb muscles of infected mice at 4 days postinfection, but undetectable at 8 days. In contrast, viral RNA levels in the brains and hind limb muscles of RG/B5-wt-infected mice were much higher and increased from 4 to 8 days postinfection (Fig. 8). This result implied that RG/B5-AR only replicated at the initial time points of infection *in vivo*, and the subsequent disappearance of RG/B5-AR could be ascribed to the host’s antiviral responses and/or the viral inability to disseminate and replicate well in the older mice. Although the replication of RG/B5-AR is self-limiting, its immunogenicity was not compromised *in vivo*. Female adult AG129 mice immunized with RG/B5-AR generated strong neutralizing antibodies at 4 weeks postimmunization and protected their pups from the lethal EV-A71 challenge (Fig. 9). However, the pathogenesis and immunogenicity of EV-A71 in the immunodeficient AG129 mice are distinct from those in humans (33, 36). The virulence attenuation and uncompromised immunogenicity of RG/B5-AR will need further confirmation in other immunocompetent animals and in humans. Moreover, the genetic stability of RG/B5-AR must be evaluated because EV-A71 can quickly acquire host-adapted mutations due to its error-prone polymerase 3D. Additional mutations around the vertex of the 5-fold axis of RG/B5-AR might not only affect viral immunogenicity (32, 33) but also reduce the augmented binding affinity to sulfated GAGs of the virus (28), which is thought to be one of reasons for the attenuation of RG/B5-AR. One possible solution could be to introduce high-fidelity polymerase mutations into RG/B5-AR to increase its genetic stability and reduce its virulence reversion (20, 21).

In conclusion, an EV-A71 mutant, RG/B5-AR, with VP1-V238A,K244R at conserved sites showed dramatically reduced viral infectivity and pathogenicity, but not significantly affected viral immunogenicity. The mutant exhibited growth attenuation *in vitro* with reduced efficiency of cell entry/exit, but the attenuation was compensated by E151 in the Vero cell line, which has been approved for EV-A71 vaccine production. Moreover, the mutant acquired augmented binding affinity to sulfated glycans, which supports the possibility that viruses can be attenuated *in vivo* by trapping them with sulfated glycans to limit their dissemination and infection. Therefore, the mutant contributes to the further development of novel antiviral vaccines against EV-A71.

## MATERIALS AND METHODS

### Cells, viruses, and food dye.

Human rhabdomyosarcoma (RD; ATCC CCL-136), human SK-N-SH (ATCC HTB11), African green monkey kidney (Vero; ATCC CCL-81), and mouse NIH/3T3 (ATCC CRL-1658) expressing FLAG-tagged human SCABR2 cell lines were maintained in Dulbecco’s modified Eagle’s medium (DMEM; Life Technologies) supplemented with 10% fetal bovine serum (FBS) (DMEM-10, Biowest) and 1× antibiotic-antimycotic (Life Technologies) in a 37°C humidified incubator with 5% CO_2_. An EV-A71 clinical isolate, NUH0083 (subgenogroup B5, GenBank accession number FJ461781), was originally propagated in Vero cells and named EV-A71-B5. The food dye brilliant black BN (11220-25MG, Sigma-Aldrich) was dissolved in sterile deionized water (diH_2_O) at 10 mM and then diluted to the desired concentrations with DMEM.

### Virus propagation and purification.

RD or Vero cell monolayers with 80% to 100% confluence in T-flasks and plates (Nunc) were infected by each virus at an MOI of 0.1 to 1 and incubated at 37°C. When more than 80% of cells detached and exhibited a CPE after 2 to 3 days, the cells were freeze-thawed three times, and the supernatants containing viruses were harvested, aliquoted, and stored at -80°C for future experiments after centrifugation at 3,000 × *g* for 10 min. For virus purification, the supernatants containing virions were further centrifuged at 10,000 × *g* for 20 min at 4°C to remove small cell debris. The Twenty-milliliter aliquots of supernatants were then transferred into polycarbonate ultracentrifuge bottles (catalog no. 355618; Beckman Coulter) with 2.5 ml of 20% sucrose in PBS as cushion. The bottles were loaded into a Type 70 Ti rotor (Beckman Coulter) and centrifuged at 120,000 × *g* for 3 h at 4°C. The supernatant and sucrose were decanted as much as possible after the ultracentrifugation, and then the virus pellet in each bottle was resuspended in 2 ml of PBS or PBS containing 0.1% FBS for 2 to 6 h on ice with gentle agitation. The resuspended virions were filtered through a 0.22-μm sterile syringe filter (Millipore), aliquoted, and stored at −80°C.

### Virus inactivation by UV light.

Purified RG/B5-wt or RG/B5-AR virion samples suspended in PBS in 35- by 10-mm cell culture dishes (Nunc) were inactivated by UV light in a biosafety class II cabinet (NuAire) for 30 min on a heating block cooled by ice. Complete inactivation was defined by no CPE after 3 blind passages of the samples in Vero cells.

### 50% tissue culture infective dose.

TCID_50_ assay was carried out in cultured cells using the Reed and Muench formula as previously described (35). Briefly, serial 10-fold dilutions of each virus sample were transferred into octuplicate wells in 96-well flat-bottomed microtiter plates (Nunc) in which 10^4^ RD or Vero cells were seeded. No virus was added to control wells. The plates were incubated at 37°C in the incubator, and the cells were observed daily for CPE up to 5 days postinfection.

### Plaque assay.

The assay was carried out according to a previous description (35). Briefly, serial 10-fold dilutions of virus samples were inoculated into RD or Vero cell monolayers in the presence or absence of 100 μM E151 in 6-well plates. After virus absorption at 37°C for 2 h, the monolayers were rinsed twice with PBS and overlaid with 1% UltraPure low-melting-point agarose (Thermo Scientific) in DMEM-2 with or without E151. After the agarose solidified at room temperature, the RD and Vero cells were incubated for 4 and 5 days, respectively, at 37°C. The monolayer cells were then fixed with 4% formalin in PBS and further stained with 0.1% crystal violet in diH_2_O. The plates with a ruler were imaged by a ChemiDoc Touch gel imager (Bio-Rad), and the number and diameter of plaques were analyzed by Image Lab software.

### Quantitative reverse transcription-PCR.

Total viral RNA in infected cells and mouse tissues was purified by RNeasy minikit (Qiagen), while the viral RNA genomes of virions in the supernatant were purified using the E.Z.N.A. viral RNA kit (Omega Bio-tek). Viral RNA was quantified by QuantiNova Probe RT-PCR kit using the primers qRT-PCR-EV-F and qRT-PCR-EV-R and a TaqMan probe, qRT-PCR-EV-probe. Beta-actin mRNA, an internal control, was also quantified using qRT-PCR-β-actin-F, qRT-PCR-β-actin-R, and qRT-PCR-β-actin-probe (Table 3). The RT-PCR was carried out using a Rotor-Gene Q real-time PCR cycler (Qiagen) according to a previous report (35).

**TABLE 3:** List of primers used in this experimental study

Primer name	Sequence (5′ → 3′)^*a*^
B5-VP1-V238A-F	CGGACCGCCGGGTCTTCAAAGTCCAG
B5-VP1-V238A-R	AGACCCGGCGGTCCGCACCGAGAAAG
B5-VP1-K244R-F	AAGTCCCGCTACCCTTTGGTTGTC
B5-VP1-K244R-R	AGGGTAGCGGGACTTTGAAGACC
seqEV-A71-P1-F1	CCTCCGGCCCCTGAATG
seqEV-A71-P1-R1	GCGRGAGCTRTCTTCCCA
seqEV-A71-P1-F2	GTGGGAAAAGTCATCCAAG
seqEV-A71-P1-R2	CATCGTGTCTCAATCATACTC
qRT-PCR-EV-F	AATAAATCATAACCTCCGGCCCCTGAATG
qRT-PCR-EV-R	AATAAATCATAAGAAACACGGACACCCAAAGTAGTC
qRT-PCR-EV-Probe	[6-FAM] TCCGCTGCAGAGTTRCCCGTTACGA [TAMRA]
qRT-PCR-β-actin-F	AATAAATCATAACCBTCCTTCYTGGGYATGGA
qRT-PCR-β-actin-R	AATAAATCATAAGAGGAGCRATGATCTTGAT
qRT-PCR-β-actin-Probe	[HEX] TCCATCATGAAGTGYGACGTBGACATCCG [TAMRA]

a 6-FAM, 6-carboxyfluorescein; TAMRA, 6-carboxytetramethylrhodamine; HEX, 6-carboxy-2,4,4,5,7,7-hexachlorofluorescein.

### Western blot.

Viral and cellular proteins were dissolved in Laemmli SDS loading buffer, separated by 12% SDS-polyacrylamide gel, and transferred onto nitrocellulose membranes with a pore size of 0.2 μm (Bio-Rad). The membrane was first blocked by 5% nonfat milk in PBS with 0.05% Tween 20 (PBST), and Western blot analysis was performed as previously described (34). To detect the VP1 protein of EV-A71, the membranes were blotted with mouse monoclonal antibody 1D9, followed by horseradish peroxidase (HRP)-conjugated goat anti-mouse IgG antibody (P0260; Dako). To detect cellular beta-actin, the membranes were blotted with HRP-conjugated mouse anti-human beta-actin monoclonal antibody (sc-376421; Santa Cruz). To detect GST and GST-tagged CypA, the membranes were blotted with mouse anti-GST serum followed by HRP-conjugated goat anti-mouse IgG antibody. To detect the human Fc recombinant proteins SCARB2 and CTLA-4, the membranes were blotted with HRP-conjugated rabbit anti-human IgG antibody (P021402-2; Dako). After rinsing with PBST, the membranes were incubated with Clarity Western ECL substrate (Bio-Rad). Chemiluminescent signals were recorded and quantified by a ChemiDoc Touch gel imager and Image Lab software (Bio-Rad), respectively.

### Immunofluorescent assay.

The viral VP1 antigen in EV-A71-infected or viral gnomic RNA-transfected cells was detected as previously described (34). Briefly, the cells were fixed, permeabilized, washed, and subsequently incubated at 4°C with guinea pig anti-VP1 serum for at least 3 h with gentle agitation. After washing with PBST, the cells were incubated overnight with AF488-conjugated anti-guinea pig IgG antibody (1:1,000; Life Technologies). The cells were then stained by Hoechst 33258 in PBS at a concentration of 1 μg/ml for 10 min. After another 3 PBST washes, the cells in PBS were observed under a UV microscope (Olympus).

### Selection and sequencing of EV-A71-B5 mutants.

E151-sensitive EV-A71-B5 was serially passaged in Vero cells in the presence of 10 μM E151 10 times and then in the presence of 100 μM E151 another 4 times. The fourteenth passaged virus, named Vero/B5-E151-P14, was selected, as it was E151-enhanced in Vero cells. The genomic RNA of EV-A71-B5 and Vero/B5-E151-P14 was extracted using the E.Z.N.A. viral RNA kit (Omega Bio-tek), and the P1 genes were amplified by one-step RT-PCR kit (Qiagen) using the primers seqEV-A71-P1-F and seqEV-A71-P1-R. The RT-PCR thermal cycling conditions were applied at an initial incubation at 50°C for 30 min (reverse transcription), 95°C for 15 min (initial PCR activation step), followed by 40 cycles: 94°C for 30 s (denaturation), 58°C for 30 s (annealing), and 68°C for 4 min (extension), and a final extension at 68°C for 10 min. The PCR products were purified after gel electrophoresis and inserted into pJET1.2 vector (Thermo Scientific) for sequencing. The P1 sequences were compared, and the mutations responsible for E151 enhancement were further confirmed by reverse genetics.

### Construction and generation of EV-A71 mutants.

The extracted EV-A71-B5 genomic RNA was amplified by RT-PCR and then put under the human RNA polymerase I promoter as previously described (47), and the infectious cDNA clone was named pJET-EV-A71-B5. The mutations VP1-V238A and VP1-K244R were introduced into pJET-EV-A71-B5 by site-directed mutagenesis with the corresponding primers (Table 3) according to the In-Fusion protocol (TaKaRa). The reverse genetics EV-A71-B5 wild-type virus and mutants were generated by direct transfection of their corresponding infectious plasmids into Vero cells. Each plasmid transfection was carried out in duplicates, and then the medium was replaced by fresh DMEM-10 for one or by DMEM-10 with 100 μM E151 for the other at 6 h posttransfection. After 3 days, the transfected cells were freeze-thawed thrice, and the viruses in the supernatant were diluted 10 times and further propagated in Vero cells for another two passages in the presence or absence of 100 μM E151. The accuracy of the VP1 genes of the second passage viruses was confirmed by sequencing. All RG viruses used in subsequent experiments were obtained from a stock of the second passage.

### Virus growth in the presence of different concentrations of E151.

The viruses EV-A71-B5, Vero/B5-E151-P14, RG/B5-wt, and RG/B5-AR were diluted to a concentration of 2 × 10^4^ PFU/0.5 ml in DMEM-10 with E151 concentrations ranging from 0 to 100 μM and incubated for 1 h at 37°C. Vero cells were seeded into 24-well plates at a density of 2 × 10^5^ cells per well and incubated for 6 h in the incubator, and then the medium was replaced by 0.5 ml of virus diluents so that the cells were infected at an MOI of 0.1 PFU/cell. At 24 h postinfection, the viral protein, RNA, and progeny were quantified by Western blotting, qRT-PCR, and TCID_50_, respectively.

### One-step growth kinetics of EV-A71.

Vero cell monolayers in 24-well plates were infected with RG/B5-wt or RG/B5-AR at an MOI of 5 or 0.01. After 1 h of incubation at 37°C, infected cells were rinsed thrice with PBS to remove unbound viruses and further cultured with DMEM-10 in the incubator. Viral VP1 protein, RNA, and progeny were quantified at 1, 8, 16, 24, 32, 40, and 48 h postinfection.

### Cell entry bypass assay.

RD or Vero cells were seeded into 24-well plates at a density of 10^5^ cells per well and incubated overnight at 37°C. Copies of purified genomic RNA (2 × 10^7^) of RG/B5-wt or RG/B5-AR were transfected into cells by Lipofectamine 2000 (Life Technologies). For comparison, 2 × 10^7^ virions of RG/B5-wt or RG/B5-AR were inoculated into parallel wells. The cells were washed twice with PBS and cultured with fresh DMEM-10 after 3 h of transfection or infection. The viral VP1 antigen, RNA, and extracellular and intracellular virions were then quantified by immunofluorescent assay (IFA) and qRT-PCR at 10 h postinfection or transfection when one life cycle of EV-A71 finished.

### Titration of extracellular and intracellular viruses.

RD or Vero cells were seeded into 24-well plates at a density of 10^5^ cells per well, incubated overnight at 37°C, and then infected with 500 μl of RG/B5-wt or RG/B5-AR at an MOI of 0.01 in the presence or absence of 100 μM E151. At 48 h postinfection, almost all cells exhibited CPE. For each infection sample, cell culture medium from infected wells was transferred into a centrifuge tube, and 250 μl of fresh medium was added into the wells. After centrifugation at 3,000 × *g* for 10 min at 4°C, the supernatant containing extracellular viruses was transferred into a fresh tube, while the cell pellet was resuspended with 250 μl of fresh medium and transferred back to the wells. The infected cells in wells were freeze-thawed three times to release the intracellular viruses into the medium, and the supernatant was collected after the centrifugation. The virus samples of RG/B5-wt and RG/B5-AR were then titrated in Vero cells in the absence and presence of 100 μM E151, respectively.

### Binding of EV-A71 to sulfated glycans.

HS (H7640), CS (C4384), and DS (D4911), all purchased from Sigma, were first linked to epoxy-activated agarose lyophilized powder (E6632; Sigma). A 0.5-g aliquot of the powder resin was soaked and washed three times with double-distilled water and suspended in 5 ml of distilled water. Each sulfated glycan (5 mg) dissolved in 1 ml of coupling buffer (0.1 M Na_2_CO_3_ and 0.15 M NaCl [pH 13]) was mixed with 0.5 ml of the suspended resin at room temperature overnight for coupling reaction. The resin was then washed with PBS five times and incubated with 1 ml of quenching buffer (1 M ethanolamine [pH 8]) at room temperature for 6 h. The resin was further washed three times with acetate buffer (0.1 M CH_3_COONa and 0.5 M NaCl [pH 4), followed by Tris buffer (0.1 M Tris-HCl and 0.5 M NaCl [pH 8]), and stored in 0.5 ml of PBS with 0.02% NaN_3_ at 4°C for the following experiments.

Sulfated GAG-conjugated or negative-control resin (100 μl each) was blocked by PBS containing 1% bovine serum albumin (BSA) at 4°C overnight and washed twice with PBS. It was then mixed with 10^7^ RG/B5-wt, RG/B5-V238A, RG/B5-K244R, or RG/B5-AR virions in 1 ml of binding buffer (20 mM Tris-HCl, 150 mM NaCl, 0.2% Igepal CA-630, and 0.1% BSA [pH 7.4]) and incubated at room temperature for 2 h with gentle agitation. After incubation, the resin was washed 3 times with PBST, and the bound virions were quantified by qRT-PCR.

### Attachment of EV-A71 to EDTA detached cells.

RD and RD-XT2^−/−^ cells (35) in a T-flask were first detached with PBS containing 5 mM EDTA, washed three times with PBS, and then and resuspended in DMEM-10. After being chilled on ice for 10 min, 10^6^ cells were incubated with 2 × 10^8^ ice-cold RG/B5-wt or RG/B5-AR virions at 4°C for 1 h. Unbound virions were removed by rinsing the cells three times with ice-cold PBS; total RNA of attached virions and cells was then purified using RNeasy minikit (Qiagen). The viral RNA and cellular beta-actin mRNA were quantified by qRT-PCR.

### Binding of EV-A71 to uncoating factors.

Purified virions of RG/B5-wt or RG/B5-AR (10^9^) were first diluted in 500 μl of PBS+ (PBS, 0.2% Igepal CA-630, and 0.1% BSA [pH 7.4]), and then mixed with 50 μl of BSA-pretreated protein A/G Sepharose beads (Santa Cruz) containing 3 μg of SCARB2-Fc (R&D systems) or CTLA-4-Fc (R&D systems) or with 50 μl of BSA-blocked glutathione Sepharose beads (Thermo Fisher) containing 30 μg of GST-CypA or GST (34). After incubation at 4°C for 1.5 h with gentle agitation, the beads were washed three times with ice-cold PBS containing 0.01% Tween 20, and the bound proteins and viral VP1 antigen were analyzed by Western blotting.

### Ethics statement.

Mice were housed in individually ventilated cages inside an animal biosafety level 2 (ABSL2) laboratory for animal care and use. All animal experiments were carried out in accordance with the Guides for Animal Experiments of the National Institute of Infectious Diseases (NIID), and experimental protocols were reviewed and approved by the Institutional Animal Care and Use Committee (IACUC) of the Temasek Life Sciences Laboratory Ltd., Singapore. The IACUC-approved project was “TLL-18-016: Pathogenicity and immunogenicity of drug dependent human enterovirus 71 variants in AG129 mouse.”

### Determination of virulence of RG/B5-wt and RG/B5-AR.

AG129 mice (129/Sv mice without interferon alpha/beta/gamma receptors) breeders were purchased from B&K Universal (United Kingdom). Usually, one male and two females were housed in a ventilated cage for breeding and observed daily. After 3 days from the birth of pups, the mother and pups were transferred into a new cage for later experiments.

Groups of AG129 mice (*n* = 6 to 8) at 5, 7, 11, and 14 days old were first intraperitoneally infected by 10^8^ virions of purified RG/B5-wt or RG/B5-AR, and their survival rates and clinical scores were then recorded for 3 weeks as previously described (34). As the RG/B5-AR failed to kill 7-day-old and older mice, determination of the LD_50_ of both viruses were further carried out in 5-day-old mice. Mice were intraperitoneally inoculated with 50 μl of 10-fold serial dilutions of RG/B5-wt (10^5^ to 10^8^ virions) or RG/B5-AR (10^7^ to 10^9^ virions) or with PBS, and their survival rates and clinical scores were recorded for 3 weeks.

### Quantification of viral RNA in mouse organ/tissue.

To study the replication and dissemination of RG/B5-wt and RG/B5-AR in 5-day-old mice, the mice were intraperitoneally infected with 10^6.25^ virions of RG/B5-wt (∼3 LD_50_) or RG/B5-AR. At 4 and 8 days postinfection, hind limb muscles and brains (*n* = 6 to 8) were harvested, frozen, and weighed after euthanasia of the mice. Total RNA purification of mouse samples was performed using a RNeasy minikit (Qiagen). Briefly, about 50 mg of the samples was homogenized in 1 ml of Buffer RLT containing 1% β-mercaptoethanol using a TissueLyser LT homogenizer (Qiagen). After centrifugation of the lysates at 16,000 × *g* for 3 min, 100 μl of the supernatant of each sample were transferred into a new Eppendorf tube by pipetting for RNA extraction in duplicate. The purified RNA was eluted by nuclease-free water, and the amounts of viral RNA and mouse beta-actin mRNA were quantified by qRT-PCR.

### Virus adsorption by mouse organ/tissue.

Mouse brains, hind limb muscles, hearts, lungs, kidneys, and intestines were collected and homogenized using the TissueLyser LT in DMEM-2. Insoluble fractions of homogenates were washed with DMEM-2 three times, and 100 mg (wet wt) was incubated with 10^6^ virions of RG/B5-wt or RG/B5-AR in 1 ml of DMEM-2 at 4°C for 1 h with gentle agitation. After centrifugation at 3,000 × *g* for 20 min, nonadsorbed virions in supernatants were subjected to plaque assays for virus quantification. The number of input virions minus that of nonadsorbed virions equals the number of adsorbed virions.

### Lethal EV-A71 challenge of suckling mice from immunized adult AG129 females.

AG129 females (10 to 14 weeks old; *n* = 5) were intraperitoneally immunized with 300 μl of 10^9^ live RG/B5-wt virions, 10^9^ live RG/B5-AR virions, 10^9^ UV-inactivated RG/B5-AR virions, or PBS. At 4 weeks postimmunization, the sera were collected for EV-A71-neutralizing antibody titration. The females were then used for breeding to produce pups. The suckling pups were intraperitoneally challenged by 100 LD_50_ of homologous RG/B5-wt strain (5.6 × 10^7^ virions) at 5 days old or by 10 LD_50_ of heterologous EV-A71-C1 strain (1.5 × 10^8^ TCID_50_ in RD cells) at 14 days old (34), and their survival rates and clinical scores were recorded up to 3 weeks.

### Neutralizing antibody titration.

The sera were heat-inactivated in a 56°C water bath for 30 min and 2-fold serially diluted by DMEM-2 in 96-well plates. Diluted sera (50 μl) were mixed with 50 μl of DMEM-2 containing 100 TCID_50_ of RG/B5-wt or EV-A71-C1 and then incubated at 37°C for 1 h. A 50-μl aliquot of DMEM-10 containing 10^4^ Vero cells was then inoculated into 96-well plates, and the plates were incubated at 37°C up to 5 days for CPE development. Each serum sample was duplicated, and its neutralizing antibody titer were defined as the reciprocal of the highest dilution fold which completely prevented CPE caused by virus infection in both wells. Every sample was used in two independent experiments, and the average value is represented.

### Analysis of viral protein sequence and structure.

Viral nucleotide and protein sequences were analyzed using the Lasergene 13 package (DNASTAR). The crystal structure data of 3VBS (EV-A71) were obtained from the Protein Data Bank. Molecular graphics and analyses of proteins and virions were done using UCSF Chimera.

### Statistics.

All quantification of viral amounts and binding assays was performed in duplicates or triplicates. Their values were compared using Student’s *t* test, analysis of variance (ANOVA) test, or nonparametric Mann-Whitney test in Excel (Microsoft) and/or Prism version 8.0.1 (GraphPad Software, USA). Kaplan-Meier survival curves and mean clinical score curves were analyzed with GraphPad Prism using the log-rank test and the Wilcoxon test, respectively. Two-tailed *P* values of <0.05 were considered statistically significant (*, *P* < 0.05; **, *P* < 0.01; ***, *P* < 0.001).
